# The promising role of new molecular biomarkers in prostate cancer: from coding and non-coding genes to artificial intelligence approaches

**DOI:** 10.1038/s41391-022-00537-2

**Published:** 2022-04-14

**Authors:** Ana Paula Alarcón-Zendejas, Anna Scavuzzo, Miguel A. Jiménez-Ríos, Rosa M. Álvarez-Gómez, Rogelio Montiel-Manríquez, Clementina Castro-Hernández, Miguel A. Jiménez-Dávila, Delia Pérez-Montiel, Rodrigo González-Barrios, Francisco Jiménez-Trejo, Cristian Arriaga-Canon, Luis A. Herrera

**Affiliations:** 1 Unidad de Investigación Biomédica en Cáncer, Instituto Nacional de Cancerología-Instituto de Investigaciones Biomédicas, UNAM, Avenida San Fernando No. 22 Col. Sección XVI, Tlalpan. C.P., 14080 CDMX, México; 2 Departamento de Urología, Instituto Nacional de Cancerología, Avenida San Fernando No. 22 Col. Sección XVI, Tlalpan. C.P., 14080 CDMX, México; 3 Clínica de Cáncer Hereditario, Instituto Nacional de Cancerología, Avenida San Fernando No. 22 Col. Sección XVI, Tlalpan. C.P., 14080 CDMX, México; 4 Departamento de Patología, Instituto Nacional de Cancerología, Avenida San Fernando No. 22 Col. Sección XVI, Tlalpan. C.P., 14080 CDMX, México; 5 Instituto Nacional de Pediatría, Insurgentes Sur No. 3700-C. Coyoacán. C.P., 04530 CDMX, México; 6 Instituto Nacional de Medicina Genómica, Periférico Sur 4809. Arenal Tepepan, Tlalpan. C.P., 14610 Mexico City, Mexico

**Keywords:** Prognostic markers, Prostate cancer

## Abstract

**Background:**

Risk stratification or progression in prostate cancer is performed with the support of clinical-pathological data such as the sum of the Gleason score and serum levels PSA. For several decades, methods aimed at the early detection of prostate cancer have included the determination of PSA serum levels. The aim of this systematic review is to provide an overview about recent advances in the discovery of new molecular biomarkers through transcriptomics, genomics and artificial intelligence that are expected to improve clinical management of the prostate cancer patient.

**Methods:**

An exhaustive search was conducted by Pubmed, Google Scholar and Connected Papers using keywords relating to the genetics, genomics and artificial intelligence in prostate cancer, it includes “biomarkers”, “non-coding RNAs”, “lncRNAs”, “microRNAs”, “repetitive sequence”, “prognosis”, “prediction”, “whole-genome sequencing”, “RNA-Seq”, “transcriptome”, “machine learning”, and “deep learning”.

**Results:**

New advances, including the search for changes in novel biomarkers such as mRNAs, microRNAs, lncRNAs, and repetitive sequences, are expected to contribute to an earlier and accurate diagnosis for each patient in the context of precision medicine, thus improving the prognosis and quality of life of patients. We analyze several aspects that are relevant for prostate cancer including its new molecular markers associated with diagnosis, prognosis, and prediction to therapy and how bioinformatic approaches such as machine learning and deep learning can contribute to clinic. Furthermore, we also include current techniques that will allow an earlier diagnosis, such as Spatial Transcriptomics, Exome Sequencing, and Whole-Genome Sequencing.

**Conclusion:**

Transcriptomic and genomic analysis have contributed to generate knowledge in the field of prostate carcinogenesis, new information about coding and non-coding genes as biomarkers has emerged. Synergies created by the implementation of artificial intelligence to analyze and understand sequencing data have allowed the development of clinical strategies that facilitate decision-making and improve personalized management in prostate cancer.

## Introduction

Detection of prostate cancer (PCa) includes the measurement of PSA serum levels and the digital rectal exam (DRE), contributing with the detection of PCa in early stages. The localized disease, when it is confined to the prostate, is treated with radical prostatectomy (RP) [1]. In contrast, localized advanced PCa is treated with surgery, adjuvants, radiotherapy such as external beam radiation or brachytherapy; and hormone therapy such as Luteinizing hormone-releasing hormone (LHRH) agonists and antagonists, abiraterone, and enzalutamide; while metastatic disease is usually treated with hormone therapy such as apalutamide, and chemotherapy [2]. If PCa is diagnosed on time, the treatment can be effective and with minimal morbidity [3]. In order to cover the proportion of indeterminate findings, novel diagnostic biomarkers have been developed such as Prostate Health Index (PHI), 4 K score, SelectMDx, ConfirmMDx and PCA3 [4]. Although several studies have been performed to analyze different molecular biomarkers, such as variant V7 of the androgen receptor [5] or inactivation of the *PTEN* or *c-MYC* gene [6], to date, none of them have been approved as a prognostic biomarker for use in clinical settings [7, 8]. Currently, there are a few molecular prognostic biomarkers in clinical use such as OncotypeDX Genomic Prostate Score [9], Prolaris [10], ProMark [11], and Decipher [12], based on cancer-associated gene panels [4]. These molecular tests guide the urologist to establish the appropriate treatment and predict recurrence and progression risk after localized treatment. However, it is important to keep searching for molecular markers that can aid in early diagnosis and prognosis of the patient as well as the establishment of patient response to different treatments, such as new genes, gene fusions, AR variants and non-coding RNAs.

At present, there is an intense debate regarding PSA as a diagnostic, prognostic and screening tool in PCa, and therefore it is especially important to focus on other types of molecular markers that can support clinical outcomes and decision making for therapy [13]. In particular, transcriptome and genomics analysis have contributed to generate new knowledge in the study of PCa and the intracellular signaling pathways that regulate prostate carcinogenesis generating new information about its biology [14]. Otherwise, artificial intelligence and some of its algorithms have been served for clinical application in monitoring, detection, diagnosis, and treatment to generate new clinical predictive models to PCa Management [15]. Alternatively, several studies have combined histology with genomic data, integrating omics information with pathological images in PCa [16, 17] and with implementation of artificial intelligence algorithms such as deep learning and machine learning have served to establish a connection from different branches of omics to get clinical prediction models, thus, creating an integrative perspective that facilitates the discovery of new diagnostic, prognostic and therapeutic molecular biomarkers. Finally, the importance of precision medicine and the fusion between sequencing and artificial intelligence is established with the aim of creating synergies that allow the development of more specific and advanced systems that facilitate obtaining relevant clinical strategies for decision-making and personalized management of PCa patients.

## Methods

Aiming to search for new molecular biomarkers involved in the diagnosis, prognosis and prediction, an exhaustive search was conducted by Pubmed, Google Scholar and Connected Papers using keywords relating to the genetics, genomics, transcriptomics and artificial intelligence in PCa, it includes “biomarkers”, “non-coding RNAs”, “lncRNAs”, “miRNAs”, “repetitive sequence”, “risk”, “prognosis”, “prediction”, “therapy”, “exome”, “whole-genome sequencing”, “RNA-Seq”, “transcriptome”, “artificial intelligence”, “machine learning”, and “deep learning”.

### Biomarkers and precision medicine in prostate cancer

**Fig. 1:**
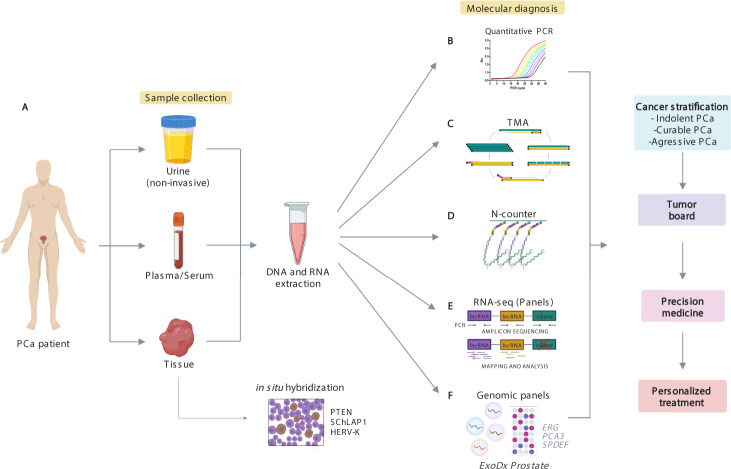
Landscape of precision medicine and molecular tools in prostate cancer. **A** Sample collection. This can be achieved by sampling tissue, blood or even urine (a non-invasive sampling) from the patient and proceeding with a direct detection of the biomarker by in situ hybridization in the tissue sample or a nucleic acid extraction and a molecular assay. **B** Quantitative PCR (qPCR). This molecular tool can be used to quantify gene expression by determining the amount of a target sequence present in the sample based on fluorescent emission, such as My Prostate Score [119]. **C** Transcription Mediated Amplification (TMA), PROGENSA is a current test based on a TMA assay. **D** N-counter. It is a highly multiplexed single-molecule counting system where two probes are used to target the RNA molecule of interest, a capture probe and a reporter probe. Dong et al. used the NanoString nCounter assay to target mRNA transcripts in EVs from PCA cell lines [120]. **E** RNA Sequencing. The RNA massive sequencing allows analyzing the entire transcriptome and even transcripts yet to be discovered. **F** There are also genomic panels used for the diagnostic of PCa focused on specific biomarkers, such as the commercial test ExoDx Prostate [121] that detects the expression levels of *ERG, PCA3 and SPDEF* by qPCR in exosomes from urine samples.

The National Cancer Institute of the United States of America defines a biomarker as a biological molecule that can be detected in blood, tissue, or bodily fluids that can be measured and whose values allow the identification of a normal or abnormal process, as well as a disease [18]. From the large variety of molecular markers currently in existence, they can be classified according to the clinical context for which they will be used. For example, there are diagnostic, prognostic, and predictive molecular biomarkers [19]. This process has led to the era of precision medicine where the selection of treatment is based on the molecular characteristics of the tumor of each patient [20] (Fig. 1). In the following paragraphs, we will mention and describe some molecular biomarkers that have recently been reported as useful in PCa patient management, including coding and non-coding genes.

### Coding genes as molecular markers of prostate cancer under clinical investigation

Several novel biomarkers for PCa have been proposed, however, their clinical utility remains to be discussed. Nevertheless, coding genes used as biomarkers such as AR, BRCA2, PTEN and the gene fusion TMPRSS2-ERG have predictive value for treatment response and are used in clinical practice [21–23]. Although these molecular markers offer valuable prognostic information for clinical practice, they are only functional in a subset of patients and more clinical trials are needed to validate their utility (Table 1).

**Table 1:** PCa-associated biomarkers approached by clinical trials.

Biomarker	Clinical trial phase	Type of cancer	Patients included	Clinical trial ID
TMPRSS2-ERG	Phase II	mCRPC and recurrent PCa	148	NCT01576172
	Phase II	Recurrent PCa, stage IV PCa	29	NCT00330161
	Phase II	Prostatic adenocarcinoma	148	NCT01682772
	Phase I	Advanced or metastatic PCa	113	NCT00749502
	Phase I	High risk PCa	65	NCT02588404
	Phase I	Localized or locally advanced PCa, biochemical recurrent PCa	84	NCT03421015
	Phase II	High risk PCa	208	NCT02573636
TP53	Phase III	mCRPC	750	NCT03903835
	Phase I/II	Prostatic neoplasia	36	NCT00900614
	Phase III	Localized PCa	7 776	NCT00001469
	Phase I	Localized or locally advanced PCa, biochemical recurrent PCa	84	NCT03421015
AR	Phase I	Hormone refractory PCa	140	NCT00510718
	Phase II	PCa	45	NCT01990196
	Phase II	Recurrent PCa	42	NCT03311555
	Phase I	mCRPC	58	NCT01516866
	Phase II	Metastatic PCa, CRPC	60	NCT04090528
	Phase I	PCa	40	NCT02411786
	Phase II	mCRPC	8	NCT02379390
	Phase II	Biochemical recurrent PCa	90	NCT01790126
	Phase II	Advanced hormone dependent PCa	90	NCT01861236
BRCA2	Phase II	High risk PCa	100	NCT02154672
	Phase III	mCRPC	408	NCT03075735
	Phase III	Genetic predisposition to PCa	1 700	NCT00261456
	Phase II	mCRPC	40	NCT04038502
	Phase II	mCRPC	70	NCT03012321
	Phase III	mCRPC	387	NCT02987543
PTEN/P13K/AKT/mTOR	Phase II	High risk PCa	208	NCT02573636
	Phase III	CRPC	120	NCT03580239
	Phase II	PCa previously treated	108	NCT01251861
	Phase I	PCa previously treated with enzalutamide	36	NCT03310541
	Phase I	Stage III and IV PCa	62	NCT01480154
	Phase II	mCRPC	9	NCT02091531
MGMT	NA	NA	NA	NA
DNMT1	Phase I	mCRPC	19	NCT05037500
	NA	Prostatic adenocarcinoma	19	NCT01118741
	Phase III	Prostatic adenocarcinoma	80	NCT03535675
	Phase I	Adenocarcinoma of the Prostate, Recurrent PCa, Stage I, IIA, IIB, III and IV PCa	32	NCT01912820
	Phase I/II	Prostate Carcinoma	NA	NCT03709550
JMJD3	NA	NA	NA	NA
KDM4B	NA	NA	NA	NA
CDK9	Phase I	Castrate Resistant Prostate Cancer	100	NCT05159518
SF3B2	NA	NA	NA	NA
AR-V7	Phase III	Castrate Resistant Prostate Cancer	953	NCT02438007
AR-V3	NA	NA	NA	NA
HDAC6	NA	NA	NA	NA
PRUNE2	NA	NA	NA	NA
Circulating tumor cells	NA	Prostate Cancer Obesity	67	NCT02453139
	Phase II	Patients with PSA 4–10 ng/mL	500	NCT03488706
	Phase II	Localized PCa	200	NCT01961713
	Phase II	mCRPC	11	NCT00887640
	Phase II	Advanced PCa	24	NCT02552394
	Phase II	mCRPC	140	NCT03050866
	Phase I	PCa	60	NCT02450435
cell-free DNA	Phase III	Metastatic PCa	1038	NCT00134056
	Phase I	PCa	12	NCT04081428
	Phase II	Metastatic PCa	300	NCT02853097
	Phase II	PCa	68	NCT02941029
	Phase II	PCa	30	NCT03284684
Extracellular vesicles	NA	PCa	108	NCT04298398
miRNAs	Phase II	mCRPC	40	NCT02471469
	Phase II	mCRPC	46	NCT04188275
	Phase III	High risk PCa	300	NCT01220427
	Phase I	PCa	240	NCT03911999
	Phase I	PCa	60	NCT02366494
lncRNAS	NA	Prostatic neoplasia	507	NCT01024959

*mCRPC* Metastatic castration resistance prostate cancer, *CRPC* Castration resistance prostate cancer, *PCa* prostate cancer, *PSA* Prostate Specific Antigen, *NA* Not Applicable.

Information obtained from https://clinicaltrials.gov/ (Last accessed: February 25, 2021).

On the other hand, several mechanisms involved in prostate tumorigenesis such as epigenetic changes, alternative splicing, and the presence of gene variants, are possible novel biomarkers based on coding-genes with potential clinical utility [24]. For example, regarding epigenetic regulators, the coding genes *MGMT* [25], *DNMT1* [26], and *JMJD3* [27], which are involved in DNA methylation, have been associated with the risk of PCa mortality (HR 0.90; *p*-value = 3.5 × 10^2^) [25] as well as prostate tumor development (*p*-values = 0.03 and 0.05, respectively [26, 27]). Similarly, other proteins related to splicing process, such as KDM4B [28], CDK9 [29] and SF3B2 [30] have been recently associated with generation of androgen receptor variant AR-V7 (*p*-value < 0.05 [28–30]). Furthermore, the splicing variants by themselves are of particular interest for PCa research [31], androgen receptor variants [32] have been described as important factors in PCa development and prognosis, such as variant AR-V3 and its prognostic value (*p*-value = 0.05) [33]. Likewise, HRAS [34] and PRUNE2 [35] are novel variants related to PCa development with clinical utility yet to be confirmed. Therefore, research focused on finding new coding genes with clinical application as biomarkers, could improve PCa prognosis and treatment.

Another example of coding genes that may have potential clinical utility for PCa correspond to gene mutations involved in hereditary cancer, where it represents the etiology of 5–10% of all neoplasms [36], in which PCa has been associated with family history of cancer [37]. Paradoxically, few high-susceptibility genes consistently related to the hereditary of PCa have been identified, presenting a pattern of dominant autosomal inheritance [38], that has been linked to phenotypic variation and genetic heterogeneity, limiting its association with PCa predisposition [39]. Currently, the analysis of Pathogenic Variants (PV) in predisposition genes associated with defects in homologous recombination and mismatch repair [40] which represents therapeutic targets to PARP1 inhibitors and chemotherapies with platinum compounds, particularly in patients with metastatic and castration-resistant disease [41, 42]. The use of multi-gene panels in germline diagnosis has identified PV in 7% to 12% of PCa patients [43, 44], highlighting *BRCA1, BRCA2, ATM, BRIP1, CHEK2, NBN, BARD1, RAD51C, MRE11A* and *PALB2* (homologous recombination repair); *MLH1, MSH2, MSH6* and *PMS2* (mismatch repair) as high risk genes, which have clinical guidelines; option for risk reduction surgeries, and personalized treatment, which benefits the PCa patient [45] (Supplementary Table 1). Although hereditary PCa does not imply a generalized molecular diagnosis, it does entail the identification of metastatic disease; early age of onset, and cancer family history, who will have benefit for the therapeutic options and family prevention as a result of the molecular approach [46, 47].

Although all these molecular biomarkers have a potential clinical application, current clinical trials have not been able to determine whether they have sufficient sensitivity and specificity to be considered for clinical purposes, as well as all the genes discussed above are coding genes. Therefore, it is important to focus on the search for new biomarkers, like non-coding genes, which can contribute to the diagnosis and prognosis of PCa patients.

### Noncoding genes as molecular markers in prostate cancer

Most of molecular biomarkers in PCa are based in coding genes, but as previous studies have demonstrated; mRNAs tend to have less tissue- and stage-specific expression. In contrast, non-coding RNAs tend to have more tissue-specific and stage-specific expression in disease, which is one of the main reasons noncoding RNAs have been proposed as molecular biomarkers in cancer [48]. In the following paragraphs we describe some of the newest candidates as specific molecular biomarkers in PCa clinical research.

### miRNA

One of the most studied small ncRNAs are microRNAs (miRNAs), these are single stranded RNAs of 21–25 nucleotides in length that regulate the post-transcriptional degradation of messenger RNAs and inhibit their translation into proteins. Because of their high stability in body fluids [49] as well as to changes of physical and chemical conditions [50], miRNAs are interesting molecules to be used as biomarkers in cancer. Free miRNAs can be found in several bodily fluids, such as blood, urine, semen, among others [51] and their expression levels are tissue-specific and have been found to be deregulated in cancer [52]. Moreover, they exhibit differential expression between tumor and normal tissues and are useful for tumor classification according to the lineage of origin, differentiation stage, and tumor aggressiveness [53]. It has been reported that circulating miRNAs can be packed in extracellular vesicles (EV) or in association with proteins such as Argonaute2 or lipoproteins in bio-fluids including blood and urine [54–56]. Some miRNAs, such as miR-21, miR-221, miR-1290, and miR-375, have been overexpressed and associated with prognosis in CRPC patients [55, 57]. Yaman and collaborators quantified the levels of miR-21, miR-142, and miR-221 in PCa patients and reported that overexpression of these three miRNAs were associated with an advanced PCa stage [58]. Other groups have identified miRNAs in plasma and serum of patients with locally advanced and metastatic PCa, with BPH and in healthy individuals, showing that differences between each group (i.e., higher levels of miRNAs in patients with locally advanced and metastatic PCa highlight the role of miRNAs as diagnostic biomarkers [59]. Several groups have studied the diagnostic, prognostic, and predictive characteristics of miRNAs circulating in the plasma and serum of PCa patients finding differentially expressed miRNAs according to the Gleason index [60], response to treatment with docetaxel [61], and high blood PSA values [62]. In another study, a panel consisting of four miRNAs was proposed as a biomarker for the diagnosis of PCa [63]. The four miRNAs (miR-4289, miR-326, miR-152-3p and miR-98-5p) were upregulated in plasma of PCa patients compared to healthy controls and was able to differentiate between PCa patients and control individuals with an area under the ROC curve of 0.88, proving their diagnostic accuracy. In the study conducted by Sharova and collaborators [49], a circulating miRNA test consisting of measuring the level of 3 circulating miRNAs (miR-106a, miR-130b and miR-223) was proposed to differentiate between localized PCa and BPH patients. In this test two ratios are calculated: miR-106a/miR-130b and miR-106a/miR-223 ratios, the results showed a better performance (specificity: 0.806, sensitivity: 0.833, accuracy: 0.821) in comparison to PSA (specificity: 0.065, sensitivity: 0.889, accuracy: 0.507), the area under the ROC curve for miRNA test was 0.84 while for PSA was 0.56. This test could be helpful for PCa screening to avoid unnecessary biopsies and assessment of PCa risk. Indeed, the use of miRNAs as biomarkers in PCa has shown promising results for risk assessment, diagnosis, and prognosis. Implementation of miRNA-based tests in combination with gene-based biomarkers could improve the clinical management of PCa patients.

### Long non-coding RNAs

As mentioned above, RNA molecules seem to have a critical role in cancer pathways including those within PCa. Long non-coding RNAs are known to be RNA transcripts longer than 200 nucleotides with no protein-coding potential [64], these two major differences distinguish them from mRNA transcripts and any other non-coding RNA. LncRNAs have been implicated in several biological processes such as chromatin-reprogramming, genomic imprinting, transcriptional regulation in *cis* and *trans* and post-transcriptional regulation of mRNAs [65–67]. Among some pathological features in which lncRNAs are involved are cell proliferation, tumorigenesis and malignant transformation [68], this is why several studies have proposed lncRNAs as tumor-suppressor genes and oncogenes [69, 70]. Lately, lncRNAs have drawn the attention not only because of their critical role in cancer, but because of their potential as molecular biomarkers due to their tissue-specific and tumor-specific expression [68, 71]. Some lncRNAs, such as PCA3, SChLAP1, and PCAT1 have been proposed as good candidates for biomarkers mainly due to their differential expression in PCa patients [72]. PCA3 is an overexpressed PCa-specific oncogene discovered in 1999 by Bussemakers [73]. PCA3 is already considered a PCa biomarker, and it is measured by the commercial test PROGENSA approved in 2012 by the FDA [74–76] helping to reduce ~40% of unnecessary biopsies providing a great utility in urological diagnosis [77]. PROGENSA PCA3 test has a sensitivity of 62% and a specificity of 75% [78] demonstrating why lncRNAs can be one of the molecular markers with clinical utility. Similarly, SChLAP1 is known for its high expression levels in PCa. This lncRNA antagonizes the SWI/SNF complex promoting aggressiveness and metastasis of the tumor [79]. Its effectiveness as a biomarker has been proved by assays such as RNA in situ hybridization leading to the development of several tests based on the detection of SChLAP1 expression levels and linking them with the patient’s clinical-stage [80]. Therefore, SChLAP1 is considered as a promising biomarker of clinical utility and one of the best genes for prediction of metastasis and biochemical recurrence in PCa patients [79, 81]. Along with these, Luo and collaborators reported that lncRNA-p21 is overexpressed in neuroendocrine PCa (NEPC) and that a treatment based upon enzalutamide increases its expression, and thus, the neuroendocrine differentiation; all of this is caused by the alteration of the Enz/AR/lncRNA-p21/EZH2/STAT3 axis [82]. PCAT1 is another upregulated oncogenic RNA originally identified in PCa by RNA-sequencing analysis [83]. It is related to cell proliferation, apoptosis, migration, and invasion as well as epithelial-mesenchymal transition and cancer progression via the *Wnt/β-catenin* signaling pathway [84]. Finally, PCAT1 negatively regulates BRCA2 tumor suppressor protein, positively regulates Myc oncoprotein [85] and it might be also acting as a miRNA sponge involved in cell growth [83]. Hence, PCAT1 is considered as a potential biomarker for PCa prognosis and prediction, supporting the statement that lncRNAs represent potential molecular biomarkers in the management of PCa (Table 2). Most of these candidates and a large number of transcriptional units were found due to the breakthrough of the high-throughput massive sequencing technology, specifically, RNA-Seq. Finally, lncRNAs could be used in combination with gene-based biomarkers and gene fusions to increase the sensitivity and specificity of molecular diagnostic tests, which will improve clinical patient management including early detection, diagnosis, prognosis, and prediction of response to treatment [86].

**Table 2:** New biomarkers and their clinical potential in prostate cancer.

Biomarker	Type	Symbol	Validation	Reference
miRNAs	Diagnostic	let-7a, miR-145 and miR-155	Independently validated	[126]
		miR-21	Independently validated	[127]
		miR-32-5p	Independently validated	[128]
		miR-141	Independently validated	[129]
		miR-301a	Research Use Only	[130]
	Prognostic	miR-96-5p, miR-183-5p, miR-145-5p, miR221-5p	Independently validated	[131]
		miR-301a	Research Use Only	[130]
		miR-187	Research Use Only	[132]
		miR-1	Independently validated	[133]
		miRs-301a, 652, 454, 223 and 139	Independently validated	[134]
	Therapy response predictive	miR-106-b	Research Use Only	[135]
		miR-21	Independently validated	[136]
		miR-200	Independently validated	[61]
		miR-890	Research Use Only	[137]
		miR-34a	Research Use Only	[138]
lncRNAs	Diagnostic	PCA3	FDA Approved, 2012	[139–141]
		MALAT-1	Independently validated	[142, 143]
		PCAT14	Independently validated	[144]
		LOC100287482	Research Use Only	[145]
		FR0348383	Independently validated	[146]
	Prognostic	SChLAP1	Independently validated	[147]
		lncRNA-ATB	Independently validated	[148]
		FALEC	Research Use Only	[149]
		TUG1	Independently validated	[150]
		SNHG9	Independently validated	[151]
	Therapy response predictive	PCAT1	Research Use Only	[152]
		GAS5	Research Use Only	[153–155]
		NEAT-1	Research Use Only	[156, 157]
		DANCR	Research Use Only	[158]
		LOXL1-AS1	Research Use Only	[159]
Repetitive sequences	Diagnostic	HERV-K	Research Use Only	[92, 93]
		MNS16A	Research Use Only	[160]
		Y-STR loci	Research Use Only	[161]
	Prognostic	TG-PCA3 STR	Research Use Only	[162]
		CAG repeats	Research Use Only	[163, 164]
		ESR1 TA	Research Use Only	[165]
		MSR1	Research Use Only	[166]
		microsatellite instability	Research Use Only	[167]
		LINE-1	Research Use Only	[94]

### Repetitive sequences

Repetitive sequences are large quantities of repeated elements throughout the haploid genome, meaning they are repeated DNA nucleotides found more than twice in the genome that comprises about 55% of the human genome or even more [87]. Their classification can vary from author to author, and it can be based on the origin, function, structure, and genomic distribution of the DNA, but it is mainly based on the latter. The five categories are simple sequence repeats, segmental duplications, tandem repeats and satellite DNA sequences, processed pseudogenes, and transposable elements [88].

Repetitive sequences are also considered as potential molecular biomarkers in diseases like cancer because some of them are overexpressed in different types of tumors cells [89]. Genome sequencing and transcriptome sequencing have improved the discovery and detection of repetitive DNA and RNA elements that cannot be identified by classic biochemical methods [90]. Solovyov and collaborators [91] determined that RNA repetitive sequences are not fully detected when using the poly(A) protocol in RNA-seq procedure, while on the other hand, analyzing the expression of total RNA sequencing can not only identify the repetitive sequences more accurately but delimitate immune phenotypes in cancer and response to immunotherapy [91]. Among the candidates for biomarkers in PCa we can found the *HERV-K* sequence, which is highly expressed in malignant prostate tissue when comparing it with normal prostate tissue, it is considered as a possible early disease detection biomarker detected in PCa patient blood, and it can even increase PSA test efficiency [92, 93]. Moreover, *LINE-1* is a DNA sequence that encodes the RNA-binding protein ORF1p and presents an increased expression in PCa tissues. Its overexpression is associated to cancer tumorigenesis and its hypomethylation to PCa progression [94]. Although the experimental evidence regarding the importance of repeated sequences is not as abundant as other RNA biotypes, these transcripts have the potential to be considered as biomarkers in PCa, nevertheless, more studies are needed to prove its applications as biomarker for diagnosis, prognosis, and treatment management of PCa patients. The contribution of different sequencing methodologies has improved biomarker discovery in the field of non-coding transcripts.

### Importance of high-throughput massive sequencing in prostate cancer

**Fig. 2:**
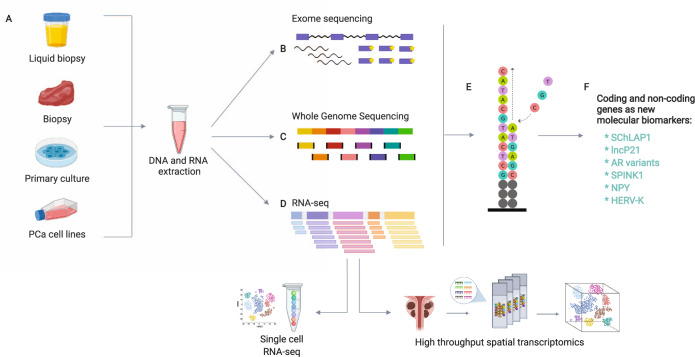
Basic research towards the discovery of new molecular biomarkers. There are several sources and molecular approaches for the detection of new biomarkers in PCa. **A** This can be achieved by using an in vivo model -for which a prostate biopsy should be taken-, a primary culture or a PCa cell line. **B** Exome sequencing. The DNA samples are first fragmented and then biotinylated oligonucleotide probes -also known as *baits*- are used to selectively hybridize to target regions in the genome. **C** Whole-Genome Sequencing. This sequencing technique allows a uniform coverage across the complete genome. **D** RNA-Seq. RNA samples are synthesized into cDNA once it has been fragmented. Then, adaptors are attached to both ends of each fragment so they can be amplificated by PCR and subsequently sequenced [122]. Within the variants of this technique can be found single-cell RNA-seq, total RNA-seq, targeted RNA-seq, small RNA-seq, spatial transcriptomics, poly-A enrichment, ribosomal RNA depletion, among others. **E** Illumina next-generation sequencing technology: Individual DNA or cDNA molecules are placed on a flowcell for sequencing by synthesis by using fluorescent labeled nucleotides. PacBio sequencer and Nanopore sequencer can read more than 100 Kb in length of DNA, as well as the disposable sequencer MinION which doesn’t need prior installation [123]. **F** After a bioinformatic data analysis the results of the sequencing provide new genes as biomarkers candidates in PCa.

DNA and RNA massive parallel sequencing has a large impact on the generation of new knowledge concerning molecular markers in cancer because it explores the whole genome and transcriptome, allowing the detection of global point mutations, insertions, deletions, variations in copy number, translocations, fusion genes, novel-transcript discovery, transcript abundance estimation, differential gene expression and differential splicing of mRNAs [95] (Fig. 2). The application of RNA-Seq provides a quantitative pattern of coding and non-coding genes with transcriptional aberrations within the cell in a disease. This technique is an emerging sequencing technology that has a promising future in disease diagnosis, prognosis, prediction and treatment [96].

Among some studies based on RNA sequencing as a potential tool for finding new PCa biomarkers and drug targets, Berglund and collaborators analyzed the heterogenicity of PCa through a spatial-transcriptomic study in which several expression profiles were identified within a tissue region obtained after RP (Gs 3 + 4, pT3b, PSA = 7.1). These expression profiles allowed the stratification of the tissue regions into cancer components or groups such as cancer, stroma, reactive stroma, normal glands and prostatic intraepithelial neoplasia (PIN). They also found specific genes as potential biomarkers within the results, for example, *SPINK1*, *PGC*, and *CPP* as specific markers of PCa (Gs 3 + 3), *NR4A1* as a specific marker of reactive stroma, and *NPY* as a specific marker of PIN. The fact that these markers are expressed in specific and different locations, demonstrates the level of heterogenicity in prostatic tumors and that studies based on RNA sequencing technologies can open the door to the discovery of novel molecular biomarkers [97].

**Fig. 3:**
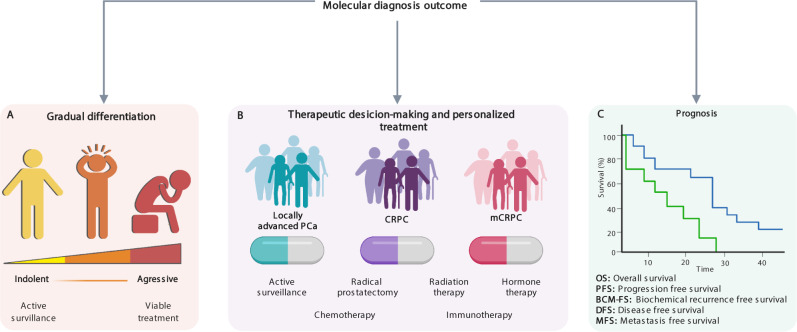
Molecular diagnosis and precision medicine in cancer patient management. The advantages of using approaches that target these signatures for disease diagnosis can be many. **A** PCa-stage discrimination. Each clinical-pathological profile will be stratified using the emerging techniques mentioned earlier, the results obtained could determine whether the patient has an indolent cancer or if it is an aggressive one. **B** Personalized treatment. The molecular diagnosis can also determine which specific treatment the patient should receive according to their molecular profile and the type of PCa they have, such as active surveillance, hormone therapy, surgery, radiation therapy, chemotherapy or immunotherapy (e.g., PD-1 inhibitors, sipuleucel-T vaccine [124]). **C** Prognosis. Finishing by getting a prognostic overview of the length of time that the patient will be alive or how well will the patient respond to the treatment he has been given. The prognosis can include overall-survival, progression-free survival, biochemical recurrence-free survival, disease-free survival, cancer-specific survival, and metastasis-free survival.

On the other hand, there is an urgent need to classify patients according to the most appropriate and effective therapy to increase the efficacy of treatment and reduce unnecessary interventions that have no effect on the patients (Fig. 3). An example of this characteristic is a study supporting the use of exomes in precision medicine has been reported by Robinson and collaborators, who demonstrated that actionable mutations detected with the aid of exomes in castration-resistant PCa patients can help determine the best treatment to use and responses of the patients. The results established a mean rate of 4.4 mutations per Mb, in addition to a gain and loss of chromosome regions, with gains in *AR* and losses in the genes *CHD1, PTEN, RB1*, and *TP53*. The relevance of this study is that the molecular changes are actionable in 90% of the samples of CRPC patients, and in particular, patients with mutations in genes such as *BRCA2* (12% of cases) and *ATM* (22% of cases) benefited from treatment with PARP inhibitors (olaparib) [98]. In another study, Armenia and collaborators identified 70 significantly mutated genes that had not been previously associated with PCa, some of them are *CUL3* (a ubiquitin ligase that function as a scaffold in the proteasome system), *SPEN* (a transcription factor involved in repression of gene expression), and *KMT2C* and *KMT2D* (epigenetic regulators with histone-lysine N-methyltransferase activity) by analyzing exome sequencing data from 1013 PCa samples [99]. The markers found in this, and other studies could be used as part of gene signatures aimed to stratifying patients with localized and metastatic PCa. Furthermore, recent whole-genome studies have identified mechanisms that generate complex chromosome rearrangements in PCa. Baca and collaborators sequenced the whole genome of 57 prostate tumors and identified several DNA translocations and deletions that arose independently during oncogenesis and progression. They called this phenomenon “chromoplexy” referring to the coordinated and considerable dysregulation of multiple cancer genes supporting a model of punctuated cancer evolution [100]. Therefore, studies based on genomics generate information that could help oncologists to predict the response to treatment, allowing more personalized and effective management of patients with advanced PCa, and considering that not only the coding proportion of the genome has this potential, the non-coding fraction of the genome should also be included. This experimental and clinical approach provides information about the emerging responses that current therapies, such as androgen deprivation, and their effect in PCa patients. Sequencing analysis can also provide the necessary data for a more specific and enriched molecular classification of PCa and could provide delineated subtypes among patients for better management [101].

### Artificial intelligence in prostate cancer research

Current methods for the detection of PCa show limitations in its detection [102], representing a need to improve PCa diagnostic and patient stratification with complementary tools. Artificial intelligence (AI) has proven to be an essential implement for clinical diagnoses, and it refers to the ability of a computational process to recognize patterns and make decisions that previously required human intellect to achieve a certain purpose [103].

**Fig. 4:**
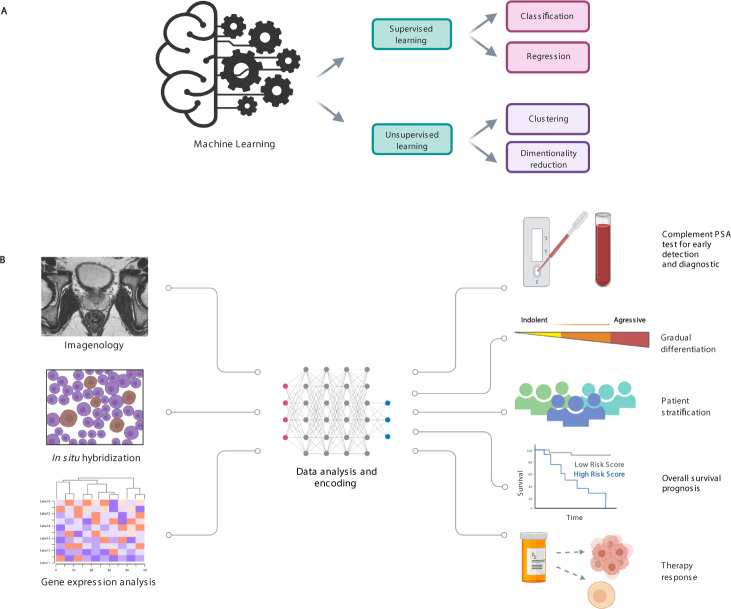
Artificial intelligence and its application in patient stratification in prostate cancer. ML is an artificial intelligence approach that can predict a possible outcome in PCa research and improve the patient management. **A** ML techniques. These algorithms are divided into two main types of learning: supervised learning and unsupervised learning. The former uses pre-determined explicit data, it is the most used in radiology and is based on classification and regression (deep learning, convolutional neural network, random forest, support vector machine, decision tree, logistic regression, among others [125]). The latter uses the features of the training data and doesn’t have a prior division of data in categories, it is based on clustering and dimensional reduction (K-means, hierarchical clustering, among others). **B** ML applied in PCa management. A recent application of ML is the prediction and analysis of radiomic data. This approach aims to improve the patient stratification and management using imageology, tissue analysis, and molecular data so the clinicians can offer a personalized treatment by differentiating the grade of the disease, stratifying the patients, and determine the therapy response.

Machine learning (ML) is a discipline that teaches computers how to build models from the massive data sets that they are assigned with and learn from them. This technologic approach is based on statistic algorithms, most of these algorithms are mathematical models that map the variables (features) of a data sample into a set of outcomes [104, 105]. Then, these algorithms go through a process of training to be able to predict the labels by analyzing the features [15]. The types of learning used in these models are mainly classified as supervised learning and unsupervised learning (Fig. 4A). Supervised learning uses explicit data sets determined by experts, the computer uses the programmed algorithms to minimize the prediction error, which is measured by the difference between the predicted labels and the known labels such as lineal logistic regression and random forest [106]. On the other hand, unsupervised learning relies on samples that are separated into different classes based on the features of the training data such as principal component analysis [106]. It has been suggested that ML could improve some aspects of biomedicine such as disease diagnosis, monitoring, anatomical imaging of organs, tissue biopsies and personalized treatment by using a collection of molecular and phenotypic data [107]. It has also been proved as useful for its application in the human genome project and advances in cancer research and management [106].

The advantageous outcome of ML also applies to PCa research by improving diagnostic and prognostic accuracy, treatment, imaging, surgical interventions, genomics and transcriptomics. It has been reported that machines can be trained to recognize complex patterns in sequencing data together with radiographic images (such as those generated from computed tomography scanning and magnetic resonance) by classifying pixels for segmentation and registration [108]. Its techniques can identify specific genes or sets of genes within expression profiles and specific expression rate that can predict a certain clinical outcome such as progression, biochemical recurrence or metastasis in PCa [109]. There are commercial genomic classifiers available, such as Decipher, that use the random forest algorithm for prediction of PCa metastasis based on the expression analysis of 22 RNA biomarkers of aggressive PCa [15, 110].

Besides, some studies have applied ML algorithms to identify and associate non-coding RNA biomarkers for PCa diagnosis such as lncRNAs [111, 112], and *s*everal reports have developed specific algorithms, such as XGBoost by Zhang and collaborators that associate lncRNAs with several cancer types [113], this algorithm is the basis of an improved method called CRlncRC2 which was found to be more sensitive and specific than his previous version CRlncRC. Moreover, miRNAs are another potential biomarker identified through ML algorithms. Bertoli and collaborators used a support vector machine model to detect 29 miRNAs for diagnostic PCa with 97% of accuracy and 7 miRNAs which can be used in prognostic of PCa with about 66% accuracy [114]*.* Another study group developed a boosted random forest-based algorithm called MEDICASCY to detect cancer drug side effects, indications, efficacy, and mode of action using the chemical structure of the drug. This algorithm showed an 80% precision for detecting drugs that can help inhibit the growth prostatic tumors, as well as ovarian and breast tumors [115].

Likewise, deep learning (DL) is a branch derived from machine learning than can be used to recognize and classify tissue structures in digital information corresponding to a pathology [116]. Tolkach and collaborators developed a trained model based on the technology of deep that recognized tumor tissue from images of 400 histological slides from different patients, as well as a novel algorithm based on three-dimensional reconstruction of PCa architecture that can improve the Gleason grading [117]. Similarly, there are other algorithms that have been applied in clinics, for example, a Support Vector Machine (SVM) model was used for the detection of positive and negative biopsies through dynamic contrast-enhanced and diffusion tensor imaging data [15]. In a 2020 DL study, a deep neural network method was used to identify AR mutations during treatment for PCa. The predictions made by the algorithm can recognize mutants that resist the inhibitor darolutamide and other mutations of pharmacological interest in PCa [118]. Therefore, the development and application of AI using ML in clinical practice could open an infinite landscape of approaches within PCa data analysis (combination of coding and non-coding genes) improving the patient management in a near future.

## Conclusions

Evidence based on clinical studies that focuses on finding new biomarkers suggests that there is a wide molecular field that lies unexplored and that could be the key for many clinical challenges nowadays. These markers, such as the coding (*AR, BRCA2, PTEN*, MLH1, CUL3, *SPEN*) and non-coding genes (PCA3, SChLAP1, *HERV-K* and miR-21), the *TMPRSS2-ERG* gene fusion including their derivatives, and the androgen receptor variant 7 can be found using genomic, transcriptomics and AI approaches. However, these are not the only alterations that can be used for the diagnostic, prognostic and prediction in the management of PCa patients. The clinical evidence mentioned in this review suggests the importance of these types of molecular markers (coding and non-coding genes) and their roles in the decision-making process for establishing the most adequate treatment for patients suffering from this disease. Thus, it is important to establish the types of actionable mutations, or their combinations, in patients with advanced PCa (locally advanced and metastatic), allowing us to determine the type of treatment that will provide a positive response in the PCa patient. In this area, genomic analysis, transcriptome sequencing, and new approaches like spatial transcriptomics, along with the clinical-pathological information, could provide the necessary information. Likewise, the application of ML algorithms will accelerate the identification and discovery of novel molecular biomarkers and it will lead biomedical investigation towards artificial-intelligence-based precision medicine, so it can improve patient management as well as their quality of life, and, in the near future, allow a scientific revolution in medicine for the management of the PCa patient. The new molecular biomarkers mentioned here along with the novel bioinformatic approaches of AI and sequencing techniques will improve biomedical research by complementing PSA test for screening, stratifying patients, and identifying new molecular biomarkers for differentiation of indolent and aggressive disease, prognostic, predictive and surrogate biomarkers with clinical utility (Fig. 4B). Finally, the fusion between sequencing and AI is established with the aim of creating synergies that allow the development of more specific and advanced systems that facilitate obtaining relevant clinical strategies for decision-making and personalized management of PCa patients to combat this global public health problem in men.

## Supplementary information

Supplementary Table 1. Main Cancer Susceptibility Genes with Therapeutic Implications for Prostate Cancer
