# Supplementary material for: The promising role of new molecular biomarkers in prostate cancer: from coding and non-coding genes to artificial intelligence approaches
Source: Prostate Cancer Prostatic Dis. 2022 Apr 14;25(3):431–43. doi: 10.1038/s41391-022-00537-2 (PMC9385485; doi:10.1038/s41391-022-00537-2)
Supplement: Supplementary file 1 — Supplementary Table 1. Main Cancer Susceptibility Genes with Therapeutic Implications for Prostate Cancer [file 41391_2022_537_MOESM1_ESM.docx]

Supplementary Table 1. Main Cancer Susceptibility Genes with Therapeutic Implications for Prostate Cancer.

| Gene | Location | Function | Hereditary Syndrome Associated | Molecular Testing | Therapeutic implications PCa |
| --- | --- | --- | --- | --- | --- |
| *BRCA1* | 17q21.31 | Homologous recombination repair. BRCA1 translocate to DNA damage sites and coordinates repair and DNA damage signaling | HBOC  (heterozygous PV) | Germline:  Guidelines-based multigene panels.  Somatic: multigene panels; HRD test. | Olaparib and rucaparib for mCRPC (FDA approved). Potentially, other PARP inhibitors like niraparib and talazoparib |
| *BRCA2* | 13q13.1 | Homologous recombination repair. Furthermore, prevents nucleolytic degradation at stalled replication forks | HBOC (heterozygous PV)  Fanconi Anemia (homozygous PV) | Guidelines-based multigene panels.  Somatic: multigene panels; HRD test. | Olaparib and rucaparib for ) mCRPC (FDA approved). Potentially, other PARP inhibitors like niraparib and talazoparib |
| *HOBX13* | 17q21 | Homeobox transcription factor that interacts with androgen receptor to jointly regulate gene expression in the prostate | Hereditary prostate cancer | Germline: G84E mutation targeted detection (certain populations) | ND |
| *ATM* | 11q22.3 | Tumor suppressor, related to DNA damage response  pathway | ATM-related disorders (heterozygous PV). Ataxia telangiectasia syndrome (homozygous PV) | Germline:  Guidelines-based multigene panels.  Somatic: multigene panels; HRD test | Olaparib for mCRPC (FDA approved).  Potentially, other PARP inhibitors like rucaparib, niraparib and talazoparib |
| *BRIP1* | 17q23.2 | Binds directly to BRCA1 BRCT for homologous recombination repair | HBOC (heterozygous PV) | Germline: Comprehensive multigene panels.  Somatic: multigene panels; HRD test | Potentially, PARP inhibitors like olaparib, rucaparib, niraparib and talazoparib |
| *CHEK2* | 22q12.1 | Tumor suppressor, DNA-damage signaling pathway. | CHEK2-related disorders (heterozygous PV).; HBOC; Li-Fraumeni syndrome (heterozygous PV). | Germline:  Guidelines-based multigene panels.  Somatic: multigene panels; HRD test | Potentially, PARP inhibitors like olaparib, rucaparib, niraparib and talazoparib |
| *NBS1 (NBN)* | 8q21.3 | Homologous recombination repair. Component of the MRN complex, critical for cellular response to DNA damage and maintenance of chromosome integrity. | HBOC (heterozygous PV)  Nijmegen Breakage Syndrome (homozygous PV). | Germline:  Comprehensive multigene panels.  Somatic: multigene panels; HRD test. | Potentially, PARP inhibitors like olaparib, rucaparib, niraparib and talazoparib |
| *RAD51C* | 17q22 | Homologous recombination repair. Required for activation of CHEK2, and cell cycle arrest in response to DNA damage | HBOC  (heterozygous PV).  Fanconi Anemia (homozygous PV) | Germline:  Comprehensive multigene panels.  Somatic: multigene panels; HRD test. | Potentially, PARP inhibitors like olaparib, rucaparib, niraparib and talazoparib |
| *MRE11* | 11q21 | Homologous recombination repair. Component of the MRN complex, critical for cellular response to DNA damage and maintenance of chromosome integrity. | HBOC (heterozygous PV).  Ataxia telangiectasia syndrome like disorder 1 (homozygous PV) | Germline:  Comprehensive multigene panels  Somatic: multigene panels; HRD test. | Potentially, PARP inhibitors like olaparib, rucaparib, niraparib and talazoparib |
| *PALB2* | 16p12.2 | BRCA2-interacting  Protein; essential component for BRCA complex formation. | HBOC  (heterozygous PV) | Germline:  Guidelines-based multigene panels.  Somatic: multigene panels; HRD test | Potentially, PARP inhibitors like olaparib, rucaparib, niraparib and talazoparib |
| *MLH1* | 3p22.2 | Mismatch repair system (MMR). MLH1 can heterodimerize with PMS2 to form MutL alpha | Lynch syndrome (heterozygous PV).  Constitutional mismatch repair deficiency syndrome (homozygous PV) | Germline:  Guidelines-based multigene panels.  Somatic:  Microsatellite instability. MMR protein immunohistochemistry (IHC). | PD−1 inhibitor pembrolizumab patients with high microsatellite instability* (FDA approved). Immune checkpoint inhibitor immunotherapy. |
| *MSH2* | 2p21-p16.3 | Mismatch repair system.  MSH2 can heterodimerize with MSH6 to form MutS alpha. | Lynch syndrome (heterozygous PV).  Constitutional mismatch repair deficiency syndrome (homozygous PV) | Germline:  Guidelines-based multigene panels.  Somatic:  Microsatellite instability. MMR protein immunohistochemistry (IHC). | PD−1 inhibitor pembrolizumab patients with high microsatellite instability* (FDA approved). Immune checkpoint inhibitor immunotherapy. |
| *MSH6* | 2p16.3 | Mismatch repair system.  MSH6 can heterodimerize with MSH2 to form MutS alpha. | Lynch syndrome (heterozygous PV).  Constitutional mismatch repair deficiency syndrome (homozygous PV) | Germline:  Guidelines-based multigene panels.  Somatic:  Microsatellite instability. MMR protein immunohistochemistry (IHC). | PD−1 inhibitor pembrolizumab patients with high microsatellite instability* (FDA approved). Immune checkpoint inhibitor immunotherapy. |
| *PMS2* | 7p22.1 | Mismatch repair system. PMS2 can heterodimerize with MLH1 to form MutL alpha | Lynch syndrome (heterozygous PV).  Constitutional mismatch repair deficiency syndrome (homozygous PV) | Germline:  Guidelines-based multigene panels.  Somatic:  Microsatellite instability. MMR protein immunohistochemistry (IHC). | PD−1 inhibitor pembrolizumab patients with high microsatellite instability* (FDA approved). Immune checkpoint inhibitor immunotherapy. |

PV: Pathogenic Variants. HRD: Homologous Recombination Deficiency. PARP: Poly (ADP-ribose) polymerase. MMR: Mismatch Repair System. FDA: Food and Drug Administration (USA).
